# Breast Cancer Tumor Microenvironment and Molecular Aberrations Hijack Tumoricidal Immunity

**DOI:** 10.3390/cancers14020285

**Published:** 2022-01-07

**Authors:** Huey-Jen Lin, Yingguang Liu, Denene Lofland, Jiayuh Lin

**Affiliations:** 1 Department of Medical & Molecular Sciences, University of Delaware, Willard Hall Education Building, 16 West Main Street, Newark, DE 19716, USA; 2 Department of Molecular and Cellular Sciences, College of Osteopathic Medicine, Liberty University, 306 Liberty View Lane, Lynchburg, VA 24502, USA; yliu@liberty.edu; 3 Department of Microbiology and Immunology, Tower Campus, Drexel University College of Medicine, 50 Innovation Way, Wyomissing, PA 19610, USA; dl998@drexel.edu; 4 Department of Biochemistry and Molecular Biology, Molecular Medicine Graduate Program, University of Maryland School of Medicine and Greenebaum Comprehensive Cancer Center, 108 N. Greene Street, Baltimore, MD 21201, USA; JLin@som.umaryland.edu

**Keywords:** antigen presentation and recognition, breast cancer, immune evasion, immune-oncological targeted therapy, tumor-infiltrating lymphocytes, hypoxia, tumor microenvironment and metabolism

## Abstract

**Simple Summary:**

Immune therapy is designed to stimulate tumoricidal effects in a variety of solid tumors including breast carcinomas. However, the emergence of resistant clones leads to treatment failure. Understanding the molecular, cellular, and microenvironmental aberrations is crucial to uncovering underlying mechanisms and developing advanced strategies for preventing or combating these resistant malignancies. This review will summarize research findings revealing various mechanisms employed to hijack innate and adaptive immune surveillance mechanisms, develop hypoxic and tumor promoting metabolism, and foster an immune tolerance microenvironment. In addition, it will highlight potential targets for therapeutic approaches.

**Abstract:**

Breast cancer is the most common malignancy among females in western countries, where women have an overall lifetime risk of >10% for developing invasive breast carcinomas. It is not a single disease but is composed of distinct subtypes associated with different clinical outcomes and is highly heterogeneous in both the molecular and clinical aspects. Although tumor initiation is largely driven by acquired genetic alterations, recent data suggest microenvironment-mediated immune evasion may play an important role in neoplastic progression. Beyond surgical resection, radiation, and chemotherapy, additional therapeutic options include hormonal deactivation, targeted-signaling pathway treatment, DNA repair inhibition, and aberrant epigenetic reversion. Yet, the fatality rate of metastatic breast cancer remains unacceptably high, largely due to treatment resistance and metastases to brain, lung, or bone marrow where tumor bed penetration of therapeutic agents is limited. Recent studies indicate the development of immune-oncological therapy could potentially eradicate this devastating malignancy. Evidence suggests tumors express immunogenic neoantigens but the immunity towards these antigens is frequently muted. Established tumors exhibit immunological tolerance. This tolerance reflects a process of immune suppression elicited by the tumor, and it represents a critical obstacle towards successful antitumor immunotherapy. In general, immune evasive mechanisms adapted by breast cancer encompasses down-regulation of antigen presentations or recognition, lack of immune effector cells, obstruction of anti-tumor immune cell maturation, accumulation of immunosuppressive cells, production of inhibitory cytokines, chemokines or ligands/receptors, and up-regulation of immune checkpoint modulators. Together with altered metabolism and hypoxic conditions, they constitute a permissive tumor microenvironment. This article intends to discern representative incidents and to provide potential innovative therapeutic regimens to reinstate tumoricidal immunity.

## 1. Introduction

Female breast cancer (BC) is the most diagnosed malignancy, with approximately 2.3 million new cases (11.7% of all cancer incidences) worldwide in 2020. That number is projected to increase to over 3 million by 2040, according to the International Agency for Research on Cancer (GLOBOCAN) estimates [1]. 

Based on the estrogen receptor (ER), progesterone receptor (PR), human epidermal growth factor receptor 2 (HER2), as well as a BC proliferation index Ki67, our understanding of BC biology reveals 4 intrinsic molecular subtypes. They include luminal A (resembling the histological phenotype: ER^+^, PR^+^, HER2^−^, Ki67^Low^), luminal B (ER^+^, PR^+^, HER^+/−^, Ki67^High^), HER2-enriched (ER^−^, PR^−^, HER2^+^), and basal-like subtype (ER^−^, PR^−^, HER2^−^) which largely resembles triple-negative BC (TNBC) and comprises approximately 15% of all BC cases [2]. The ER is found expressed in two luminal subtypes and can distinguish luminal from non-luminal malignancies [3]. Luminal A and B subtypes are characterized by their prominent activation of luminal/hormone-regulated pathways as well as proliferation/cell cycle regulators [4]. Luminal A subtype has a higher expression of luminal-related genes or proteins such as FOXA1 and lower expression of Ki67 than Luminal B [3,5]. The HER2-enriched subtype is distinguished by the high expression of HER2-related and proliferation-associated regulators such as ERBB2/HER2, insignificant expression of luminal-related genes, and negligible expression of genes related to the basal layer of the skin (e.g., keratin 5) [6]. The Basal-like subtype is characterized by high expression of Ki67 as well as keratins 5, 14, and 17, a low to undetectable expression of HER2-related genes, and unnoticeable expression of luminal-related genes [6].

The molecular subtyping helps determine the most appropriate first-line therapy. ER^+^ tumors are targeted with endocrine therapeutic agents such as tamoxifene, aromatase inhibitors, and abemaciclib; HER2 over-expressing tumors are generally treated with HER2-blocking antibodies such as trastuzumab and pertuzumab, whereas TNBC is treated with standard cytotoxic therapies and radiotherapy [7]. However, given TNBC is frequently resistant to chemotherapy and radiotherapy, one promising treatment regimen remaining is immuno-oncology therapeutics. Considering the higher mutational burden, TNBC is known to be the most immunogenic subtype. They are frequently associated with tumor-infiltrating lymphocytes (TIL) indicative of a favorable prognosis [8].

All human nucleated cells process their intracellular proteins through the proteasome system and then present the degraded peptide fragments (the epitopes) on the major histocompatibility complex (MHC)-I. This immune complex is then scrutinized by surveillance lymphocytes. Any circulating activated T lymphocytes encountering the non-self or abnormal moieties within the peptide-MHC-I complex will either directly eliminate the target cell or produce inflammatory cytokines. After recognizing a malignant antigen, a cluster of differentiated (CD)4^+^ T helper (Th) cells can secrete pro-inflammatory cytokines to recruit additional immune cells and mount an immune response, whereas CD8^+^ cytotoxic T lymphocytes (CTL) can directly destroy tumor cells by secretion of cytotoxic molecules such as granzymes leading to apoptosis [9]. A successful antitumor immune response requires the following key phases: (1) capture of tumor antigens (or epitopes) by antigen-presenting cells followed by presentation them to lymphocytes; (2) activation and expansion of CD4^+^ and/or CD8^+^ lymphocytes; (3) secretion of inflammatory cytokines by CD4^+^ lymphocytes and destruction of tumor cells by CD8^+^ lymphocytes with involvement of dendritic cells (DCs), natural killer (NK) cells, and macrophages [10,11]. However, the tumoricidal immunity can be muted by aggressive or metastasized cancer cells leading to a detrimental phase known as “immune editing” and “immune tolerance” [12]. This review aims to not only highlight the cellular and molecular immune evasion strategies occurring at various phases, but also examine emergent therapeutic modalities that may be leveraged to overcome the immuno-oncology resistance. Due to space limitations, the authors regret that some related findings cannot be discussed in this report. 

## 2. Initial Anti-Tumor Immunity Defeats Breast Neoplasm

Breast tumors are not solely masses of excessively proliferating cells. Instead, they are intermingled with a repertoire of resident and recruited non-cancerous cells such as fibroblasts, endothelial and immune cells. These cells, along with their secreted soluble factors as well as the insoluble extracellular matrix proteins collectively constitute the tumor microenvironment (TME) [13]. 

The initial combined innate and adaptive immune responses are designed to eradicate tumor growth. This early phase comprises acute inflammatory reactions in response to tumor cell recognition, the secretion of proinflammatory cytokines, and the destruction of malignant cells by innate immune cells such as NK cells, DCs, and macrophages. Upon maturation, antigen-presenting cells (APCs; predominantly DCs and macrophages) migrate to nearby lymph nodes (LN) where they recognize, internalize, digest, and then present tumor antigens at the cell surface with MHC-I or MHC-II [14,15]. Next, the epitope-bound APCs activate tumor-specific CD4^+^ Th cells and CD8^+^ CTL that migrate to the tumor site and assist in killing. The activated T cells differentiate, proliferate, and prime for effector functions that inspect cells expressing tumor-associated antigens. After the initial immune surveillance, the tumor cells are either completely eradicated or a few immune-evading clones merge [16,17]. 

## 3. Breast Cancer Reprograms Tumoricidal Immunity

Leukocytes from the innate and adaptive immune systems participate not only in initial tumoral rejection but also during tumor growth progression and metastatic spread [12,18]. In the early phase of tumor evolution, host immune factors, in particular cells of the innate immune system, play a key role in the elimination of tumor cells [19]. However, in the equilibrium phase, tumor cells are maintained in a dormant state [20]. This progresses to an immune escape when the tumor variants emerge, blunting immune recognition and establishing an immunosuppressive TME [21]. Overall, these actions are consistent with the repertoire of immunoediting whereby aggressive tumors established distinct mechanisms to evade immune surveillance, establish immune tolerance, and promote their proliferation. 

### 3.1. Aberrant Presentation of Tumor-Associated Antigens (TAA)

A crucial process for T cell recognition of tumors is MHC-binding of TAA peptides with a presentation on the surface of tumor cells or APCs. In TNBC, high levels of the MHC-II antigen presentation pathway were found to be correlated with favorable progression-free survival, reduced rate of relapse, and abundant infiltration of CD8^+^ CTL [22]. Conversely, defects in the antigen processing and presenting machinery (APM) diminish tumor cell recognition and killing by CD8^+^ CTL. Awareness of the functionality of APM is important when administering T cell-based immunotherapy protocols [23]. Furthermore, not all mutated proteins are recognized equally by T cells. For T cell recognition, neoantigens should be processed in short peptides of 9–15 amino acids. As they vary in length, only a fraction are eligible to trigger immune recognition [24].

**Figure 1:**
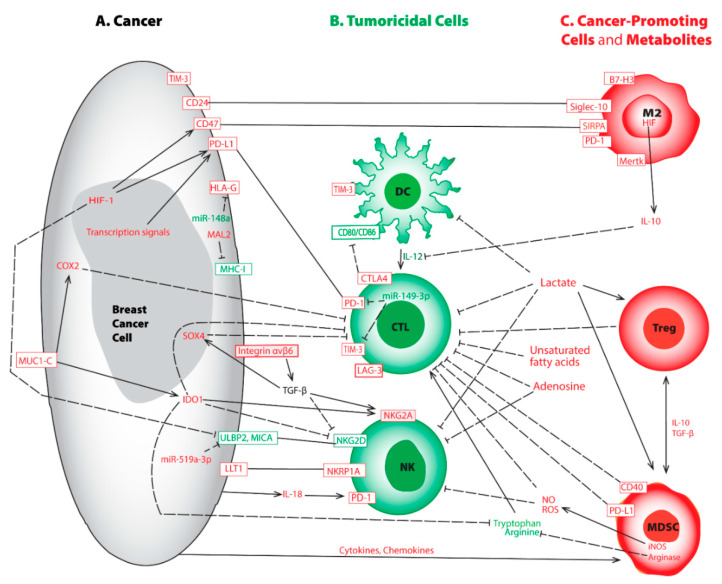
Representative aberrations in breast cancer cell (**A**), tumoricidal cells (**B**), cancer-promoting cells and metabolites (**C**) lead to immune evasion. This crosstalk map indicates the tumoricidal cells and factors (in green) and the pro-tumorigenic counterparts and factors (in red). Rectangular boxes represent cell surface molecules; plain lines indicate binding; solid arrows represent activation; and dashed lines show inhibition between modulators. Abbreviations used include cluster of differentiated (CD), CTL-associated protein 4 (CTLA-4), cyclooxygenase-2 (COX2), cytotoxic T lymphocyte (CTL), dendritic cell (DC), human leukocyte antigen G (HLA-G), hypoxia-inducible factor (HIF), indoleamine 2,3-dioxygenase (IDO), inducible nitric oxide synthase (iNOS), interleukin (IL), lymphocyte activation gene-3 (LAG-3), lectin-like transcript-1 (LLT1), M2 macrophage (M2), myelin and lymphocyte protein 2 (MAL2), major histocompatibility complex class I (MHC-I), myeloid-derived suppressor cells (MDSC), macrophage c-mer tyrosine kinase (Mertk), major histocompatibility complex (MHC), MHC-I related chain A (MICA), mucin 1 C-terminal (MUC1-C), natural killer cell (NK), nitric oxide (NO), reactive oxygen species (ROS), regulatory T cell (Treg), programmed death receptor-1 (PD-1), programmed death-ligand 1 (PD-L1), sialic acid binding Ig like lectin-10 (Siglec-10), signal regulatory protein α (SIRPA), T-cell immunoglobulin and mucin domain-containing molecule 3 (TIM-3), transforming growth factor β (TGF-β), and UL16 binding protein 2 (ULBP2). It is worth mentioning the sizes of various cells may be disproportional to their actual dimensions.

The APM is suppressed by the expression of myelin and lymphocyte protein 2 (MAL2). Initially noted in hepatoma, *MAL2* encodes a transmembrane protein associated with protein endocytosis, mainly by aiding the delivery of membrane-bound proteins and exogenous cargos from the basolateral to the apical surface [25]. Using human and mouse BC cell lines, Fang et al. demonstrated that by interacting with partner effectors, RAB7 and MHC-I molecules, MAL2 augments the endocytosis of MHC-I molecules to the late-stage endosome for degradation, downregulates CD8^+^ T cell cytotoxicity, and thus weakens immune recognition [26] (Figure 1A). High expression of MAL2 in BC decreases the stability and the level of the antigen-loaded MHC-I on the cell membrane, leading to poor antigen presentation as well as diminished cytotoxicity response from CD8^+^ CTL [26]. *MAL2*^high^ tumors escape recognition by CD8^+^ CTL cells, thereby greatly increasing immune tolerance and worsening disease prognosis [26]. In another parallel study, expression of the transport-associated proteins (TAP1/TAP2), required for proper antigen loading, is concordantly down-regulated in high-grade BC [27]. Apart from downregulating gene expression, mutations affecting antigen presentation also provide another independent mechanism of immune escape. Mutations in β2-microglobulin (B2M), a component of MHC-I, are shown to render immune suppressive effects and thereby become a potential target for therapy [28,29].

On the other hand, studies on high-grade BC demonstrate up-regulations of non-classical human leukocyte antigen (HLA)-E, HLA-F, and HLA-G are important for promoting immune escape [30,31,32]. In BC, elevated expression of HLA-G not only renders resistance towards neoadjuvant chemotherapy (NAC) [33], but also correlates with poor prognosis [33,34,35,36]. Another independent investigation on ER^+^ BC demonstrates that estrogen signaling silences a microRNA known as miR-148a that further elevates HLA-G expression and promotes immune evasion [37] (Figure 1A; Table 1). HLA-G conveys suppressive effects on adaptive and innate immunity by interacting with immune cell inhibitory receptors such as leukocyte immunoglobulin-like receptors B1 and B2 (LILRB1 and LILRB2) or killer cell immunoglobulin-like receptor 2DL4 (KIR2DL4) [38].

### 3.2. Dysfunctional CD8^+^ Tumor-Infiltrating Lymphocytes (TIL) 

NAC-induced tumor cell death results in increased TIL largely due to the release of processed antigens from dead tumor cells followed by APC uptake and presentation [39]. Among lymphocytes, CTL is associated with a favorable prognosis, and high frequencies (>60%) of TIL are predictive for therapy response to NAC [40]. Recent studies among BC subtypes demonstrate TNBC often harbors the highest numbers of TIL and they are associated with neoantigens. However, the intra-tumoral CD8^+^ CTL may present at the exhausted phase due to prolonged exposure to immune-suppressive molecules in the TME [17,41,42]. Exhausted T cells neither produce antitumor cytokines nor execute their tumoricidal functionality [43,44]. Bagati et al. demonstrate, via the integrin αvβ6–TGF-β–SOX4 signaling pathway, SOX4 expression is upregulated by the integrin αvβ6 receptor on the surface of TNBC tumor cells thereby changing transforming growth factor (TGF)-β from a latent precursor to an active form [45] (Figure 1A; Table 1). High expression of SOX4 not only blocks tumoricidal function normally associated with CTL but also correlates with a poor prognosis [45]. Antibody-mediated blockade of integrin αvβ6 attenuates SOX4 expression and restores T cell-mediated cytotoxicity [45]. Likewise, SOX4 pathway inhibition prevents the emergence of MHC-I^Low^ tumor cells that are also refractory to CD8^+^ CTL [45]. 

In addition, mounting evidence indicates interferon (IFN)-γ signaling, when chronically activated in tumors, conveys an immune-suppressive TME [46,47]. Notably, the oncogenic protein mucin 1 C-terminal (MUC1-C) is aberrantly produced by TNBC, and its expression leads to both depleted and dysfunctional CTL [48,49]. Molecular studies reveal MUC1-C induces the IFN-γ→JAK1→STAT1→IRF1 signaling cascade as well as the downstream indoleamine 2,3-dioxygenase (IDO)1 and cyclooxygenase (COX)2 effectors, thereby synergistically inhibiting CD8^+^ T cells in TNBC [42] (Figure 1A; Table 1). Upregulation of IDO1 in TNBCs lowers the levels of tryptophan, an amino acid essential for proper T-cell proliferation and immune function in the TME [50] (Figure 1A). Similarly, elevated COX2 expression in TNBCs increases the production of prostaglandin (PG) E2 leading to T-cell dysfunction [51,52]. Targeting MUC1-C could be of clinical importance by disrupting this immunosuppression niche [42]. 

### 3.3. Aberrant Immune Checkpoint Modulators 

Immune responses are controlled by a plethora of checkpoint regulators that act as “security brakes”, to terminate immune reactions when an infection is resolved, to promote self-tolerance, and to protect against autoimmunity. Tumors exploit such immune checkpoint molecules, attempting to dampen antitumor responses, favor immune tolerance, and escape recognition and destruction [53]. Blocking the activity of one or several of these immune checkpoint molecules is shown to rescue otherwise exhausted antitumor T cells and, most importantly, to improve clinical outcomes as well as survival benefits in cancer patients [54]. 

#### 3.3.1. CTLA-4

At the anti-tumor immune surveillance stage, T lymphocyte activation requires recognition of peptide-loaded MHC-I by the T-cell receptor (TCR) followed by binding between CD28 on T-lymphocytes and the counterpart ligands CD80/CD86 on APCs. However, in BC, neoantigen recognition may result in the emergence of an inhibitory receptor known as CTL-associated protein 4 (CTLA-4), which is translocated to the cell surface. CTLA-4 is a homolog of CD28, and it harbors a strong affinity for binding CD80/CD86. This interaction not only hijacks the activation signal normally executed by CD28 binding but also unleashes a contrary signal that abrogates CTL function [55] (Figure 1B, Table 1). By triggering a negative feedback loop and weakening the immune surveillance effect, CTLA-4 is widely recognized as a crucial regulator of T cell self-tolerance and immune evasion leading to poor prognosis [56,57].

#### 3.3.2. PD-1 and PD-L1

Programmed death receptor-1 (PD-1) is an inhibitory transmembrane protein expressed on T cells, B cells, macrophages, and NK cells. The interaction between PD-1 and its ligand programmed death-ligand 1 (PD-L1) on tumor cells, ignites a “stop eating me” signal that directly contributes to immune evasion via promotion of peripheral T effector (Teff) cell exhaustion and conversion to immunosuppressive T regulatory (Treg) cells, thereby hindering tumor destruction [53,58] (Figure 1A,B; Table 1). PD-L1 expression ranges from 20% to 50% in all BC subtypes and is higher in TNBC patients as compared to non-TNBC [59,60,61]. High levels of PD-L1 are associated with poor overall survival (OS) [62], and elevated PD-L1 expression is involved in immune evasion and poor prognosis in TNBC [63]. 

Importantly, responses to checkpoint immunotherapy could be modulated by expression levels of PD-L1 on tumor cells. For instance, PD-L1 can be palmitoylated in its cytoplasmic domain, and this lipid modification sustains PD-L1 stability by preventing ubiquitination and subsequent degradation. Inhibition of palmitoylation shortens the lifespan of PD-L1 and enhances T cell-mediated tumoricidal activity [64] (Table 1). This finding is substantiated by Nouri et al. who elucidated the Hippo pathway effector, yes-associated protein, and transcriptional co-activator with PDZ-binding motif (YAP/TAZ) is critical in mediating anaplastic lymphoma kinase (ALK)-induced up-regulation of PD-L1 in multiple cancer cell lines. Knocking down YAP/TAZ impedes ALK-mediated immune evasion due to lowered PD-L1 expression [65]. Moreover, in a human BC stem cell model, elevated PD-L1 correlated with promoter CpG de-methylation and aberrant posttranslational histone modifications comprising lowered occupancy of repressive histones in the PD-L1 promoter region and overexpression of histone acetylation enzymes [66]. Further studies on the stem cell-enriched fraction of TNBC reveal that elevated PD-L1 expression can be induced by the Wingless/int1 (WNT) signaling pathway [67]. In addition, BRD4, a member of the bromodomain and extra-terminal domain, can transcriptionally up-regulate PD-L1 expression by binding to its promoter [68,69].

It is worth noting that in TNBC, the signal transducer and activator of transcription (STAT)-3 and its homolog STAT1 are also involved in regulating PD-L1 expression. Mechanistic studies show that phosphorylated STAT1 binds phosphorylated STAT3 in the cytoplasm, and the complex translocate into the nucleus where this heterodimer binds the PD-L1 promoter and activates its transcription [70] (Table 1). Initially, syntenin1 was shown to induce T cell exhaustion in vivo. Further studies linked STAT3 with syntenin1 and demonstrated the syntenin1-driven, STAT3-dependent signaling cascade, can upregulate PD-L1 in a TNBC model [71]. Taken together, it is rationalized that targeting syntenin1, in conjunction with inhibiting STAT3, could become a potential strategy for restoring CTL activity and thereby improving the prognostic outcomes of patients with TNBC. Additional studies demonstrate the expression of PD-L1 can be elevated by Crk in TNBC [72], by neuromedin U (NmU) in HER2^+^ BC [73], or by exposure to radiation [74].

Given the PD-1/PD-L1 association inactivates T cells and attenuates tumoricidal effects, disrupting PD-1/PD-L1 binding may evolve into a promising therapeutic regimen. As of March 2019, the United States Food and Drug Administration (FDA) had approved seven immune blockade therapies for treating a variety of cancers including BC. The antibodies primarily target two major immune checkpoint pathways, PD-1, and PD-L1, as well as CTLA [75,76]. Accordingly, the 1st line immune checkpoint blockade therapy in combination with chemotherapy was administered for treating TNBC patients who express PD-L1 [77]. For patients who respond poorly or develop secondary resistance to immunotherapy, treatments will combine with additional epigenetic agents [78,79], or with chemotherapeutic agents [80]. Current investigations are underway to determine the benefits of combining immunotherapy with radiation [81,82,83].

#### 3.3.3. LAG-3

Lymphocyte activation gene-3 (LAG-3) is a member of the immunoglobulin superfamily that was first identified in 1990 [84]. LAG-3 is expressed on activated CD4^+^ and CD8^+^ T cells and on a subset of NK cells. It is structurally similar to CD4 and binds MHC-II with a higher affinity than CD4 [85,86]. Although LAG-3’s mechanism of action remains to be elucidated, it is certain that increased expression of LAG-3 can be triggered in chronically activated T cells [87,88]. LAG-3 exerts a remarkable synergy with PD-1 to transduce the inhibitory impact on activated CD8^+^ T cells (Figure 1B). This leads to immune escape [89,90,91] through the interaction of LAG-3’s ligands available in the TME [92,93]. LAG-3 and PD-1 have been shown to be co-expressed on TIL, and blockade of both regulators had synergistic effects on restoration of the anti-tumor CD8^+^ T cell response [90,91] (Figure 1B; Table 1).

#### 3.3.4. TIM-3

T-cell immunoglobulin and mucin domain-containing molecule 3 (TIM-3), initially identified based upon its expression on CD4^+^ Th1 cells and CTL [94], is another immune checkpoint modulator that contributes to immune suppression in BC [95]. TIM-3 hampers proliferation attenuates the production of effective cytokines and augments apoptosis of effector T cells, through interaction with its ligands galectin-9, high mobility group protein B1 (HMGB1), carcinoembryonic antigen-related cell adhesion molecule 1 (CEACAM-1), and phosphatidylserine [95,96,97]. TIM-3 is expressed on a variety of immune cells including T lymphocytes, DCs, and BC cells [98] (Figure 1A,B).

Upon studying DCs, blockade of TIM-3 by introducing anti-(α)TIM-3 antibodies improves response to paclitaxel chemotherapy in murine models of luminal B and TNBC diseases [99]. Furthermore, through prolonged exposure to IL-12, combined efficacy not only boosts the effector function of intertumoral CTL but also elevates granzyme B expression with minimal cytotoxicity [99] (Table 1). Another independent study shows TIM-3 inhibits the production of the chemokine CXCL9/10 by DCs thereby limiting antitumor immunity in mammary carcinomas [100]. TIM-3 blockade, similarly, enhances response to paclitaxel, augments uptake of extracellular DNA by DCs through an endocytic process, and renders re-activation of the cytoplasmic DNA-binding by HMGB1 as well as sensing by the cyclic GMP-AMP synthase (cGAS) and stimulator of IFN genes (STING) pathway in DCs [100]. Together, upon TIM-3 blockade, elevated chemokines released from DCs can strengthen T cell effector function and response to chemotherapeutic treatment [99,100]. The immune surveillance function of DCs within tumors is emerging as a critical determinant of an effective T cell response [101], and TIM-3 inhibition depicts not only chemotherapeutic susceptibility but also promising efficacy in cancer immunotherapy. 

On the other hand, TIM-3 overexpression in BC cells promotes cell proliferation, migration, invasion, and enhances chemoresistance to paclitaxel through the overly activated NF-κB/STAT3 pathway [102]. STAT3 signaling displays a plethora of roles in immune cells and promotes the immunosuppressive function in the TME [103]. TIM-3 overexpression in BC cells activates the STAT3 signal pathway that promotes crosstalk between cancer and immune cells [104]. TIM-3 also destroys tight junctions, which further accelerates cancer progression [102]. Conversely, downregulation of TIM-3 in BC cells inhibits their proliferation, migration, invasion, and promotes apoptosis [105]. 

One of the microRNAs, miR-149-3p, has been reported to bind 3′UTRs of mRNAs encoding PD-1, TIM-3, and other immune checkpoints [106]. Treatment of CTL with miR-149-3p mimic rescues T-cell exhaustion, downregulates PD-1 and TIM-3, and thereby promotes the killing of 4T1 mouse breast tumor cells [106] (Table 1). Moreover, TIM-3 has recently emerged as a promising target for cancer immunotherapy, because it is a non-redundant regulator that differs from other better-characterized checkpoints. Several prospective studies and clinical trials have been launched in solid tumors [107]. αTIM-3 partially reverses this exhausted phenotype, results in improved expression of IFN-γ, and suppresses tumor growth in multiple preclinical models [108]. αTIM-3 antibodies have revealed successful efficacious treatment synergy when combined with αPD-1 [109] or when it is used subsequently in αPD-1 refractory cancers [110]. 

Mounting evidence demonstrates blocking one immune checkpoint can result in the upregulation of alternative modulators which potentially synergizes T cell exhaustion [111] and gives rise to compensatory mechanisms for immune evasion [111,112]. As a result of these studies, there are several antibodies against TIM-3 (e.g., TSR-022/Cobolimab, MBG453, LY3321367, BMS986258) being evaluated in clinical trials, mostly in combination with additional agents abrogating PD-1 and PD-L1 pathways [100]. Hence, future innovative BC treatment regimens may comprise TIM-3 co-blockage with additional checkpoint modulators, through the delivery of a blockade antibody cocktail or with miR-149-3p, in conjunction with non-immune-based protocols such as chemotherapy. 

### 3.4. Dichotomic Roles of Natural Killer (NK) Cells

During normal immune surveillance, NK cells and CTL express NKG2D receptors to detect malignant or damaged cells via recognition of a cellular stress response-induced ligand surge [113]. The NKG2D/NKG2DL axis renders cytotoxic activity and induces anti-tumor cytokines [114] (Figure 1B). 

However, NK cell function is also modulated by various activating and inhibitory receptors interacting with their respective ligands on target cells. For instance, Lectin-like Transcript-1 (LLT1, CLEC2D, OCIL) is a ligand on BC that interacts with NK cell receptor NKRP1A (CD161) leading to inhibition of NK cell-mediated cytolysis [115] (Figure 1A,B). Blocking LLT1 with antibodies or knocking down of the gene *LLT1* impedes this interaction and enhances the destruction of TNBCs by NK cells [116] (Table 1). Similarly, NK cells from invasive cancers express lowered NKG2D, and yet, elevated inhibitory receptors (one of which is NKG2A) due to a mechanism modulated by immunosuppressive cytokines (e.g., TGF-β and IDO1) in the TME [117] (Figure 1A,B). Another independent mechanism conveying immune escape is weakened antibody-dependent cell-mediated cytotoxicity, thus constraining antitumor effects [118]. Furthermore, interleukin (IL)-18, present in the TME, can upregulate PD-1 expression on NK cells resulting in a profound immune-suppressive outcome [119] (Figure 1A,B). 

Moreover, microRNAs produced by some tumor cells combat immune surveillance by influencing neighboring metastatic sites, adjacent immune cells, or the TME. MiR-519a-3p reduces tumor destruction by NK cells in two ways. It downregulates the ligands for NKG2D, UL16 binding protein 2 (ULBP2), and MHC-I related chain A (MICA), on the surface of cancer cells leading to obstructed recognition by NK cells [120] (Figure 1A,B). Furthermore, miR-519a-3p weakens the expression of target genes such as tumor necrosis factor-related apoptosis-inducing ligand-receptor 2 (TRAIL-R2), caspase-7, caspase-8, and granzyme B which are crucial for the apoptosis cascade [120]. Taken together, miR-519a-3p protects BC cells from NK cell-mediated destruction and increases resistance to apoptotic death, and thus synergistically contributing to immune evasion [120] (Table 1).

### 3.5. Elevated Regulatory T Cells (Treg) Suppress Immune Response and Promote Tumor Growth

As opposed to the CD8^+^ CTL, the prognostic role played by CD4^+^ T lymphocytes is of lesser weight because its subpopulation is quite heterogeneous. It comprises at least four distinct lineages, namely Th1, Th2, Th17, and Tregs, each with unique and at times opposing functions [121]. Tregs, with FOXP3 as the specific and reliable marker, normally act to suppress T-cell responses following the resolution of infections and are crucial for preventing autoimmune diseases. However, tumors can divert this modulatory mechanism and elevate the numbers of Tregs [122]. 

Apart from playing important roles in maintaining homeostasis, Tregs facilitate BC proliferation, immune evasion, and metastasis, through the production of protumorigenic cytokines and expression of immunomodulatory receptors [123,124] (Figure 1C). As such, the abundance of Tregs in breast tumor biopsies is linked to poor relapse-free survival, OS, and prognosis [125,126]. Moreover, RUNX3, a CD8^+^ lineage-specific transcription factor, was shown to bind FOXP3-promoter, enhance its transcription, and increase Treg population in the TME leading to worsened prognosis [127,128,129]. The association of intratumoral Tregs in advanced BC is explained by the upregulation of Treg-attracting chemokines in tumor cells [125,130,131], with concomitant induction of inflammatory mechanisms promoting tumor metastasis [132]. Acting as predictive biomarkers, the balance between intratumoral CTL and Treg populations greatly influences the outcome of clinical responses after neoadjuvant chemotherapies [133,134] as well as a pathological complete response [135]. 

### 3.6. Polarizing Anti-Tumor Macrophage (M1) to Tumor-Promoting Macrophages (M2)

Monocytes are often recruited to the TME in response to stimuli with subsequent differentiation to macrophages via crosstalk with cytokines and chemokines [136]. With this unique plasticity and influence by external cues, macrophages are the key elements orchestrating various aspects of TME immunity. They participate in innate as well as adaptive immunity, during both the anti-tumor phase and pro-tumoral immune evasion. Macrophages within early neoplastic tissues are frequently tumoricidal. Yet, prolonged exposure to the TME bestows them with protumorigenic properties. This suggests that macrophage plasticity may be therapeutically exploited to restore initial tumoricidal properties [137]. 

Under the influence of microenvironmental signals, macrophages can be polarized into two immunologically distinct subsets, M1 (classically activated, anti-tumoral) and M2 (alternatively activated, pro-tumoral) [138]. M2 produces various tumor-promoting factors with the most prominent being vascular endothelial growth factor (VEGF), IL-6, IL-10, and TGF-β [139,140] (Figure 1C). Notably, Ham et al. report that exosomes (the membrane-bound extracellular vesicles) released by BC cells can skew macrophage polarization toward the M2 phenotype partially via gp130/STAT3 signaling [141]. While M1 macrophages are largely involved in the milieu of normal immune reactions, prolonged exposure to a poorly vascularized TME stimulates macrophages to upregulate hypoxia-inducible factors (HIF)-1α and HIF-2α. This provides metabolic adaptation to an oxygen-poor environment and polarizes macrophages towards M2 to execute immunosuppressive functions [142]. 

M2 are predominantly marked by CD163 and account for the majority of tumor-associated macrophages (TAM). They also play pleiotropic roles in establishing a TME favorable for tumor growth, metastatic spreading, invasion, migration, angiogenesis, and secretion of soluble mediator cytokines and chemokines [143,144]. Studies in mouse models of BC show TAMs impair CD8^+^ CTL activation and proliferation through an IL-10-dependant manner. Briefly, IL-10 inhibits the production of IL-12 by dendritic cells that subsequently suppresses tumoricidal actions exerted by CTL [145] (Figure 1B,C). 

Immune checkpoint regulators in the TME provide “stop eating me” signals to hinder phagocytosis. TAMs not only express PD-1 [146,147] to evade killing by CTL, but also produce signal regulatory protein α (SIRPA) to block phagocytosis after binding with CD47 receptors on the surface of tumor cells [148,149] (Figure 1A,C). On the other hand, mounting evidence indicates that TAMs express high levels of Sialic Acid Binding Ig Like Lectin (Siglec)-10 while tumors overexpress CD24 (Figure 1A,C). Genetic ablations of Siglec-10 or CD24 and antibody blockade of the CD24–Siglec-10 complex greatly enhance phagocytosis [150]. These data highlight the role of CD24 as an anti-phagocytic signal and demonstrate its therapeutic potential [150]. CD24-deficient cells were also significantly more sensitive to CD47 blockade than normal control cells, suggesting the cooperativity of combinatorial blockade of CD24 and CD47 [150]. Dual treatment with CD24 and CD47 blocking antibodies demonstrates a vigorous induction of phagocytosis [150] (Figure 1A,C; Table 1). In a parallel context, macrophages present in mammary tumors undergo a substantial reduction of MHC-II expression mediated by tumor-expressed macrophage migration inhibitory factor (MIF). This subsequently inhibits antigen presentation and hinders adaptive immune induction [151,152]. 

B7-H3 is another nonredundant immune checkpoint modulator expressed in tumor cells, tumor vascular endothelial cells, macrophages, and other APCs. It has been widely studied in the context of tumor progression and immune evasion [153]. B7-H3 expression is elevated in TAMs of TNBC patients and strongly correlates with poor prognosis. B7-H3^high^ TAMs exhibit great pro-metastatic and immunosuppressive activity by remodeling extracellular matrix (ECM) and expanding tumor angiogenesis, thereby enhancing tumor cell dissemination, and reducing T-cell infiltration into the TME [154] (Figure 1C). B7-H3 inhibition by antibodies exerts a detrimental effect on TAM, as well as the TME, and thus, restricts tumor growth [155] (Table 1). 

Likewise, another independent M2 receptor known as macrophage c-mer tyrosine kinase (Mertk), was shown to correlate with a poor prognosis due to its capacity to sustain an immunosuppressive environment [156] (Figure 1C; Table 1). Blockade of Mertk function on macrophages decreased efferocytosis and altered the cytokine milieu. Similarly, Mertk-knockout mice or administering anti-Mertk neutralizing antibodies altered the cellular immune profile, resulting in an inflamed tumor environment with enhanced T-cell infiltration into tumors and improved cytotoxicity [156]. 

### 3.7. Myeloid-Derived Suppressor Cells (MDSC)

At the conclusion of normal hematopoiesis, immature myeloid progenitor cells (IMCs) usually differentiate into mature granulocytes, monocytes, or DCs which play essential roles in host defense against invading pathogens. However, in pathologic circumstances such as cancers, IMCs fail to proceed to a typical differentiation but rather acquire features of immature and dysfunctional myeloid populations known as myeloid-derived suppressive cells (MDSC) [157]. Circulating MDSC in peripheral blood of BC patients is elevated in all stages of the disease and positively correlated with high cancer grades as well as an extensive metastatic burden [158].

Reprogrammed by breast tumors, MDSC not only creates a tolerogenic environment by inducing Treg and blocking CTL function as well as proliferation, but also directly drives tumor growth by promoting angiogenesis, epithelial-to-mesenchymal transition, stemness, and metastasis [157,159,160,161]. MDSC can alternatively differentiate into TAM that are immune-suppressive and sustain cancer stem cell characteristics [162]. Yet, their primary action is through deterioration of innate and adaptive immune tumoricidal responses [161,163]. MDSC release arginase that depletes L-arginine from the TME and cripple T cell function, as well as produce reactive oxygen species (ROS) and nitric oxide (NO) that impede immune cell signal transduction [164] (Figure 1C).

Via a paracrine feedback loop, T lymphocytes secrete IFN-γ that plays a crucial role in augmenting MDSC in a breast tumor-bearing mice model [165]. These MDSC express CD40 and PD-L1 to attenuate the antitumor response of T cells [166,167,168] (Figure 1C). Notably, inflammatory cytokines such as PGE2, IL-6, and IL-1β, commonly produced from chronic inflammatory responses or released from tumors, can increase MDSC [169,170,171,172,173,174]. These findings depict an important linkage between chronic inflammation and tumor progression. 

Indoleamine 2,3-dioxygenase (IDO), the enzyme catalyzing oxidative cleavage of tryptophan to *N*-formylkynurenine, can lower tryptophan in the TME and exacerbate antigen-specific tolerance in T cells [175]. IDO is often released from tumors and is responsible for recruiting MDSC and fostering an immune-suppressive TME [175] (Figure 1A,C). Furthermore, TNBC secretes a variety of cytokines and chemokines, such as granulocyte colony-stimulating factor (G-CSF) and granulocyte-macrophage colony-stimulating factor (GM-CSF), to promote MDSC development [159,176,177]. Their underlying molecular mechanism was recently revealed by Li et al. [177]. Their study reports excessive aerobic glycolysis in TNBC orchestrates a molecular network leading to elevated expression of liver-enriched activator protein (LAP) that efficiently increases G-CSF and GM-CSF expression [177]. In TNBC, the effective recruitment of MDSC to the primary tumor and metastatic sites also requires another set of chemokines, CXCL2, and CCL22, that are activated by a transcription factor **Δ**Np63 produced by tumors [178] (Figure 1A,C). Together, these findings identify innovative and non-redundant options via eradicating MDSC for treating such a devastating malignancy.

## 4. Tumor Stroma, Mediators, Chemical Components, and Physical Factors Constitute the Tumor Microenvironment (TME)

The theory of cancer cell plasticity hypothesizes that the ability of tumors to adapt their phenotypes or functions is important for controlling disease progression, metastatic spread, immune evasion, and treatment outcomes [179]. A plethora of regulators produced by cancer cells, immune cells, and cancer-associated stroma (CAS) not only constitute the TME, but are also critical for coordinating cancer plasticity and immune escape. For example, elevated circulating IL-9 levels in BC patients were shown to enhance atypical anti-tumor immunity [180]. Contrarily, IL-18 present in the TME can upregulate PD-1 expression on NK cells, resulting in an immune-suppressive phenotype [119].

Mounting evidence indicates tumor stroma participates in nearly all stages of carcinogenesis and exclusion or dysfunction of CD8^+^ T cells and is correlated with an abundance of immune-suppressive cells, including M2, MDSC, Tregs, and CAS [181]. Furthermore, syndecan-2, secreted by CAS and released into the TME, can induce TGF-β signaling and upregulate CXCR4 and PD-L1 expression leading to deteriorated immune surveillance [182]. Conversely, inhibition of syndecan-2 not only attenuates TGF-β signaling, impedes PD-L1 expression, tumor growth, and metastasis, but also elevates immunotherapy susceptibility in breast tumors [182] (Table 1).

### 4.1. Dysregulated Cytokines and Chemokines

Produced by immune cells, CAS, and tumor cells, soluble mediators comprise a variety of growth factors, cytokines, and chemokines. Several of them are reported to closely participate in tumor progression, invasion, and immune escape. These include tumor necrosis factor alpha (TNF-α), transforming growth factor beta (TGF-β), insulin-like growth factor 2 (IGF-2), vascular endothelial growth factor (VEGF); cytokines IL-1, IL-4, IL-6, IL-8, IL-10; and chemokine (C-X-C motif) ligand 1 [183]. 

BC tumors engage a paracrine loop and crosstalk with the TME to suppress T-cell infiltration and function. By releasing an extracellular regulator, galectin-3, tumor cells are capable of blocking interferon gamma (IFN-γ) in the TME followed by impeding chemokines CXCL9, CXCL10, and CXCL11 leading to obstructed T-cell recruitment into the tumor bed [184] (Table 1). In addition to inactivating IFN-γ, galectin-3 conveys broad effects by binding to glycans that are associated with various glycoproteins in the extracellular matrix and forming lattices with oligomerization, as well as resulting in their inactivation. Given human cytokines are mostly glycosylated, galectin-3 secretion could depict a general strategy for tumor immune evasion and provide a potential therapeutic target. In addition, the function of CTL in the tumor bed can be inactivated by inhibitory mediators, such as IL10, IDO1, reactive oxygen species (ROS), and nitric oxide (NO) released from immune-suppressive Treg and MDSC [185] (Figure 1C), indicating synergistic networking among so-called hijacked immune cells conveys tumor-promoting outcomes.

### 4.2. Altered Signaling Pathways 

Tumor cells circumvent TIL and antigen presentation by utilizing the mitogen-activated protein kinase (MAPK) pathway. In TNBC, a lack of TIL is correlated with activated RAS/MAPK pathway [186] (Table 1). Overly activated Ras/MAPK pathway is associated with low TIL densities, impaired IFN-γ signaling, and down-regulated MHC-I and MHC-II expression, leading to reduced immune recognition. The finding was substantiated in a TNBC mouse model in which MEK inhibition upregulated the expression of cell surface MHC expression and PD-L1, both in vivo and in vitro [186]. Therefore, the dual MEK plus PD-L1/PD-1 inhibition synergized antitumor immune responses [186]. In another independent study, Franklin et al. found MEK inhibition not only affects the tumor-immune microenvironment by altering the expression of interferon-inducible MHC-I and PD-Ll expression, but also enhances immunogenicity and improves susceptibility to immune checkpoint blockade therapy [187].

The activation of IL-1 receptor family members (ILRs) along with Toll-like receptors (TLRs) constitute a critical sensing mechanism controlling tumor death or survival [188]. ILRs and TLRs activation promotes various signal transduction cascades with pro-inflammatory outcomes, including activation of NF-κB, and secretion of TNF-α. They are, in turn, necessary for NK and DC activation, along with CD8^+^ CTL priming, to combat tumor antigens [189,190]. Therefore, activation of ILRs and TLRs plays an important role in inflammation, initiation, and amplification of innate and adaptive immunity [191]. Conversely, interleukin-1 receptor 8 (IL-1R8) suppresses signaling from IL-1R1, IL-18R, and TLRs [192,193]. IL-1R8 is up-regulated during breast epithelial cell transformation and in primary BC. High IL-1R8 expression correlates with impaired innate immune sensing and T-cell exclusion, based on the immune-gene signature analysis of clinical specimens [188]. IL-1R8 expression in transformed breast epithelial cells lowered IL-1-dependent NF-κB activation as well as production of pro-inflammatory cytokines, impeded NK cell activation, and favored M2 macrophage polarization [188]. 

### 4.3. Aberrant Metabolism 

Metabolic interactions between tumors and immune cells lead to dysfunctional immunity as exemplified by exhausted T lymphocytes [194]. Mounting evidence reveals a variety of tumors actively reprogram metabolic pathways to escape tumoricidal immunity. Reports indicate glycolysis regulates T cell activation and effector function in TNBC [177]. Dysregulated metabolism in tumors is commonly linked to increased aerobic glycolysis. This important and unique phenomenon, termed the Warburg Effect, demonstrates the continued fermentation of glucose in the presence of adequate oxygen [195]. Accelerated glycolytic metabolism by the tumor shapes the TME and represents one of the profound mechanisms promoting immunosuppressive infiltrates [196,197]. Glycolytic tumors express transporters including the monocarboxylate transporter (MCT) 4 that exports lactate to the extracellular constituents. This results in an acidified TME leading to strengthened tumor invasion, metastasis, and immune evasion [198]. Accumulation of lactic acid not only blunts anticancer immunity but also increases Tregs and impairs CTL [199,200] (Figure 1C). Studies show lactate enhances the production of MDSC from the bone marrow of mice while suppressing NK cells leading to weakened cytotoxicity [201] (Figure 1C). Furthermore, lactate skews macrophages toward the tumor-promoting M2 phenotype and upregulates PD-L1 to enhance immune escape [202,203,204]. Therefore, an increased lactate concentration in the TME serves as not only a potential therapeutic target, but also a poor prognosticator [198]. 

Recent studies indicated GPR81, a G protein-coupled receptor for lactate, is upregulated in BC, and it plays both paracrine and autocrine roles to promote tumor growth through tumor-derived lactate. In the paracrine mode, the tumor-secreted lactate activates GPR81 in dendritic cells and diminishes the presentation of tumor-specific antigens to neighboring immune cells [205]. This paracrine system is complementary to the recently discovered autocrine mechanism in which lactate enhances PD-L1 expression via activation of GPR81 in tumors, thus providing an effective means for evading the immune system [206] (Table 1). As such, blocking GPR81 signaling could provide a significant enhancement of immunotherapy.

On the other hand, secretion of unsaturated fatty acids bestows another immune evasion mechanism, by inhibiting anti-tumor effects exerted by CTL [207] (Figure 1C), thereby linking obesity with a high risk of acquiring BC. Furthermore, fatty acid β-oxidation is one of the characteristics associated with M2, as well as Tregs [208,209]. Together, these phenomena emphasize the impact of altered metabolism on cancer progression.

During the course of infection or tumor progression, high levels of ATP are released into the extracellular space within the inflamed tissues or tumors [210]. Extracellular ATP acts as a proinflammatory alarm for the immune system by enhancing chemoattraction of DCs and activating monocytes and macrophages. Yet, degradation of ATP into adenosine (Ado) by murine Tregs is correlated to immunosuppression [211,212], particularly within the TME [213]. Ado is produced from sequential cleavages by enzymes CD39 and CD73 expressed by both cancer and immune cells in the TME [211,212,214]. Extracellular ATP is first converted to AMP by CD39, and the subsequent dephosphorylation of AMP to adenosine is catalyzed by CD73 [211]. Ado can favor tumor progression by impeding the cytotoxic activity of CD8^+^ CTL and NK cells through binding of receptors A2a or A2b [215,216,217] (Figure 1C; Table 1). Two crucial properties are associated with CD73^+^ T cells. First, they are largely devoid of immune checkpoints, indicating the Ado/AdoR axis is likely a revolutionized variant selected after immune checkpoint therapy. Second, these cells promote “metabolism”-driven immune tolerance. Hence, combinatorial therapy targeting non-redundant pathways Ado/AdoR axis enhances treatment efficacy [212,214,216]. 

### 4.4. Hypoxia Conveys Tumor Cell Plasticity

Due to structurally reorganized blood vessels and tumor growth exceeding the rate of vascularization, tumor hypoxia is common. These tumors have a median oxygen concentration of 1.4%, as opposed to approximately 9.3% for normal breast tissue [218]. Hypoxia plays a significant role in tumor cell plasticity. BC can be highly hypoxic, and the tumor cells located in a hypoxic TME are primarily aggressive and resistant to immunotherapy [219]. Hypoxia upregulates hypoxia-inducible factor 1α (HIF1α)-dependent ADAM10 that further induces MICA to be shed from the surface leading to poor antigen detection [220]. 

Moreover, HIF-1 stimulates CD47, a cell-surface protein that binds SIRPA on the surface of macrophages, thereby blocking phagocytosis [221] (Figure 1A,C). Interestingly, chemotherapeutic treatments cause coordinated transcriptional induction of CD47, as well as CD73 and PDL1, in TNBC cells [222]. Importantly, such an aberration can be blocked by an independent regimen of HIF-1 inhibition. This study suggests the inclusion of HIF inhibitor may prevent the unwanted countertherapeutic effects [222] (Table 1). As CD73 expression is induced by hypoxia in a HIF-dependent manner, HIF inhibition might also improve the clinical responses to treatments by blocking the Ado signaling pathway [223]. In another reversal study, under hypoxic conditions, elevated HIF-1 directly upregulates the transcription of CD47 in BC cells leading to the enhanced escape of phagocytosis by macrophages and the maintenance of CSCs [221]. HIFs have been similarly reported to promote polarizing MDSC towards M2 [224], induce Treg [225], and increase the expression of PD-L1 [167]. 

### 4.5. Extracellular Microvesicles (EVs) Modulate Immune Evasion

The communication between distant cells requires transferring of microRNAs, proteins, and lipids encapsulated in cargo-like structures known as extracellular microvesicles (EVs) [226]. EVs affect a variety of cellular and molecular pathways not only in cancers but also in other physiological and pathological conditions [227].

In cancers, EVs possess immunosuppressive properties and participate in the transfer of these traits to recipient cells. EVs are classified according to their size and the mechanisms by which they are made. Exosomes are 30–120 nm in size and are generated from the internal compartment of a cell, whereas microparticles (MPs) are 0.1–1.0 μm and produced from outer cytoplasmic membrane blebbing or shedding [228]. While exosomes play pleiotropic roles regulating the complex intracellular pathway involved in all steps of BC progression, tumor-derived microparticles (TMPs) are associated with therapy resistance [228]. For example, under hypoxic conditions, exosomes derived from two BC cell lines exert a potent immunosuppression activity by inhibiting T-cell proliferation through the TGF-β-signaling cascade [229]. Another independent study reveals BC cells’ exosomal miR-503 promotes polarization towards M2 and suppresses T-cell proliferation [230]. EVs have been shown to orchestrate a broad spectrum of immunosuppressive events at different immune response stages, including modulation of T cell proliferation and function, regulation of NK cell function, maturation of DCs, the polarization of M2 macrophages, hindrance of antibody-mediated cytotoxicity, and induction of MDSC and Tregs [231,232].

EVs released from treatment-resistant variants of HER2^+^ BC contained high levels of the immunosuppressive cytokine TGF-β1 and increased PD-L1, and were resistant to HER2 antibody trastuzumab-mediated cytotoxicity [73]. TGF-β1 in EVs is shown to contribute to immunosuppressive effects [229], and EVs from drug-resistant cells are able to disseminate the trait and raise the levels of TGF-β1 in drug-sensitive recipient cells [73]. In a parallel neoadjuvant clinical trial, the TGF-β1 level was significantly higher in EVs isolated from the serum of HER2^+^ patients who poorly respond to HER2-targeted drug treatment, as compared to those yielded from the serum of patients with partial or complete susceptibility [73]. These findings illustrate the pleiotropic function of EVs in transmitting immune-suppressive cues. As such, analyzing the constituents in EVs may uncover biomarkers important for predicting treatment efficacy [233].

### 4.6. Radiation Influences Tumoricidal Immunity

Ionizing radiation is generally used to treat a localized target, and radiotherapy plays a pivotal role in curing cancer. Ionizing radiation applied locally to the tumor site typically causes DNA damage and subsequent cell death [234]. Radiation activates several systemic biological responses, including adaptive and innate immunity that further impact tumor progression [235]. Given radiation-deceased tumor cells can stimulate dendritic and cytotoxic T-cell activity directed against viable tumor cells [236], tumors or metastatic sites distal to the initial irradiated field should, hypothetically, respond favorably to the radiation. This phenomenon is termed the “abscopal effect” [233]. However, such antitumor effects are rarely reported clinically. Instead, radiation causes not only anti-tumorigenic but also pro-tumorigenic activities, which may explain the limited response to radiotherapy.

Radiotherapy is demonstrated to induce the expression of CXCL16, one of the proinflammatory cytokines that enhances the chemotaxis of CTL cells into the tumor bed and increases their ability to execute anti-tumor cytotoxicity [237]. Contrarily, preclinical studies report macrophages exposed to radiation promote metastasis [238]. Accordingly, the elimination of macrophages from irradiated hosts attenuates tumor growth and metastasis [238]. Furthermore, Ahn et al. reported that myeloid cells in tumor-bearing mice migrate to the irradiated tumor site and accelerate tumor angiogenesis and regrowth [239]. Since pro-tumorigenic immune responses contribute to tumor re-growth, it is rationalized that blocking these unwanted reactions can improve radiotherapy outcomes. Moreover, the expression of PD-L1 on tumor cells is largely altered in response to radiation, thereby potentially contributing to the immunomodulation activity of radiotherapy [240,241]. In a TNBC model, suppressing Treg via phosphoinositide 3-kinase δ (PI3Kδ) inhibition plus immune checkpoint therapy abrogating PD-1, improves the efficacy of radiation therapy [242]. 

## 5. Conclusions

It was widely recognized that the complex interplay between immunity and tumor determines whether tumor cells will survive or be destroyed. The battle between immune surveillance and tumor-promoting activities relies on the extent to which the antitumor immune response is exerted. The main hurdle for generating a broad and robust antitumor immune response is overcoming immune escape. Tumor and immunologic constituents shed light on potential mechanisms of immune evasion in BC and the unique aspects of the TME. These include elements associated with antigen processing and presentation as well as immunosuppressive constituents, which may be targeted therapeutically. Examples of such immune-therapeutic strategies include efforts to (1) enhance CTL and NK cells; (2) boost immunostimulatory DCs; (3) improve antigen presentation; (4) block inhibitory cytokines or chemokines; (5) inhibit immune checkpoint blockades such as PD-1, PD-L1, CTLA-4, LAG-3, and TIM-3; (6) reprogram tumor-promoting M2 towards antitumor M1 macrophages; (7) differentiate Treg and MDSC to restore their tumoricidal functions; and (8) correct aberrant metabolisms (partly exemplified in Table 1). The goal of these approaches is to shift the balance and increase BC’s susceptibility to immunotherapy and other treatments. After surgical resection, rather than administering monotherapy targeting a single aberration, multiple immuno-therapy agents in conjunction with chemo- or radiation-mediated, hormone therapy-mediated, kinase-targeted, DNA repair-disrupted, aberrant epigenome-involved, and cytokine-intricated treatments, may ignite promising treatment regiments to eradicate this most common female cancer. 

**Table 1:** Representative therapeutic targets combat immune evasion of breast cancer.

**Therapeutic Agent**	**Target**	**Rationale**	**Reference(s)**
miR-148a mimic	HLA-G	Non-classical HLA promotes immune escape	[37]
avβ6 antibody	αvβ6-TGFβ-SOX4 pathway	SOX4 inhibits CTL	[45]
shRNA silences MUC1-C	MUC1-C	MUC1-C enhances IFN-γ signaling that inhibits CTL	[42,48]
αCTLA4	CTLA-4 receptor	Immune checkpoint blockade	[55]
αPD-1 or αPD-L1	PD-1/PDL-1	Immune checkpoint blockade	[58]
PD-L1 acetyltransferase inhibitor	PD-L1	Palmitoylation of PD-L1 stabilizes intracellular PD-L1	[64]
STAT1 or STAT3 inhibitor	STAT1/STAT3	The heterodimer activates transcription of PD-L1	[70]
shRNA silences Syntenin1	Syntenin1	Syntenin1 upregulates PD-L1	[71]
αLAG-3	LAG-3	Immune checkpoint blockade	[91]
αTIM-3	TIM-3	Immune checkpoint blockade	[99,100]
miR-149-3p mimic	TIM-3	miR149-3p downregulates PD-1 and TIM-3	[106]
αLLT1	LLT1	LLT1 is a tumor ligand to engage inhibitory receptors on NK cells	[116]
519a-3p antagonist	Enhances ULBP2 & MICA expression	Reinstate recognition of BC by NK cells	[120]
αCD24, αCD47, αSIRPA, αSiglec-10	CD24, CD47, SIRPA, Siglec-10	Impede bindings between BC cells and M2	[150]
αB7-H3	B7-H3	B7-H3 is an immune checkpoint modulator on tumor cells and TAMs	[155]
αMertk	Mertk	Mertk is an immunosuppressive receptor on TAMs	[156]
Syndecan 2 peptide	Syndecan-2	Syndecan-2 secreted by the stroma is immunosuppressive	[182]
Galectin inhibitor	Galectin-3	Galectin-3 produced by tumor cells inactivates multiple glycosylated cytokines and chemokines	[184]
MEK inhibitor	Ras/MAPK pathway	Overactivated MAPK pathway is immunosuppressive	[186]
GPR81 blockade	GPR81	Lactate enhances expression of PD-L1 through the GPR81 receptor	[206]
A2a inhibitor	A2a	Adenosine inhibits CTL and NK through membrane receptors such as A2a and A2b	[215,216]
HIF-1 inhibitor	HIF	HIF has broad immunosuppressive activities	[222]

The past, current, and future potential immunotherapeutic targets with respects to their rationales are exemplified. Treatment antibodies against respective target antigens are abbreviated as α. Additional abbreviations can be referred to legend for Figure 1.
